# Radial sphincterotomy in endoscopic retrograde cholangiopancreatography due to extrahepatic obstructions by large stones in the common bile duct

**DOI:** 10.20407/fmj.2020-004

**Published:** 2020-10-10

**Authors:** Kenan Yusif-zade

**Affiliations:** General Surgery Department, Military Hospital of the State Border Service, Baku, Azerbaijan

**Keywords:** ERCP, Radial sphincterotomy, Choledocholithiasis, Extra-biliary disease

## Abstract

**Objectives:**

The most common method of removal of calculi (“stones”) from the common bile duct (CBD) is an endoscopic sphincterotomy. We wished to determine the role of an improved method of sphincterotomy in choledocholithiasis: “radial sphincterotomy”.

**Methods:**

From 2017 to 2018, 54 endoscopic retrograde cholangiopancreatography (ERCP) procedures were undertaken in patients diagnosed with choledocholithiasis. Group 1 (23 patients) received a standard “pull type” sphincterotomy. The sphincterotomy incision in group 1 was made at the 11, 12 or 1 ‘O’ clock directions of a conventional clock depending on the anatomy of the papilla and stone size. Group 2 (31 patients) received a radial sphincterotomy. In this case, several incisions were made in the 11, 12 or 1 ‘O’ clock directions. The main incision was applied to the transverse fold, and other radial incisions were made below the transverse fold, without going beyond the boundaries of the proposed course of the intramural part of the CBD.

**Results:**

Stone size (mm) was classified as ≤5, 5–10, 10–15, 15–20 and >20. In group 1, the stone size was <20 mm in 21 patients, and >20 mm in two patients. In group 2, stones >20 mm were detected in seven patients, and in other cases the size was 15–20 mm. In patients who underwent radial sphincterotomy, post-ERCP pancreatitis was noted in one patient, and bleeding and perforations were not observed .

**Conclusions:**

Our method showed promising results, and deserves more extensive research and worldwide application. We recommend that only experienced endoscopists should undertake this novel procedure.

## Introduction

The most common method of calculus (“stone”) removal from the common bile duct (CBD) is an endoscopic sphincterotomy. The sphincterotomy method itself has not changed radically since its first description. During the last decade, the principles and indications have been established for endoscopic retrograde cholangiopancreatography (ERCP) and endoscopic sphincterotomy.^1,2^ However, despite a wide range of medical equipment and a considerable amount of ERCP research, intraoperative and postoperative complications remain the most acute problems.

ERCP completion is not possible in all cases. Depending on the experience of the endoscopist and anatomic parameters of the patient, success has been noted in 80% to 95% of patients.^3^ The cross-sectional size during sphincterotomy may differ in choledocholithiasis depending on the size of the stone and the anatomic structure of the papilla. A large incision during sphincterotomy leads to an increase in the prevalence of complications after ERCP, such as perforations, cholangitis, and pancreatitis. Scholars have suggested that the prevalence of mortality following ERCP for non-cancer patients is 2.2%–2.4%.^4,5^ The known data suggests that perforation rate in ERCP is about 0.56%^6^–1.6%^7^ and perforation-related mortality rate is about 20%.^8^

Removal of large stones is particularly difficult. Scholars have described methods for crushing them within the CBD by intracorporeal electro-hydraulic lithotripsy and crushing by a basket.^9^ A method for removing large-diameter stones and avoiding complications is lacking. Hence, studying and improving the methods of sphincterotomy to reduce the number of complications is a rational approach.

Previously, we assessed the relevance of a method of “radial sphincterotomy” developed by our research team.^1^ We decided to study the features of our method in more detail. The present study is a follow-up report with more cases and robust results of our previous study.^1^

Here, we investigated the role of an improved method of radial sphincterotomy. The latter comprises two or more incisions starting from one ongoing point to radial directions in the same hemisphere. We determined the prevalence and nature of complications of radial sphincterotomy and compared these data with the results of a standard sphincterotomy in ERCP.

## Methods

### Ethical approval of the study protocol

The study protocol was approved by the ethics committee of our hospital. Patients provided written informed consent to participate in this study.

### Study cohort and grouping

From October 2017 to October 2018, 54 ERCP procedures were undertaken in patients with choledocholithiasis. Stone size was taken from routine radiology reports (ultrasound and magnetic resonance imaging (MRI) examinations). This approach helped appropriate classification of the stones and adjustment of the planned sphincterotomy incisions for each patient. Stone sizes were classified as <5 mm, 5–10 mm, 10–15 mm, 15–20 mm, and >20 mm.

Two groups were created based on stone size. The group 1 (23 patients) had stones <15 mm and underwent ERCP using a standard sphincterotomy. In group 2 (31 patients), stones were >15 mm and ERCP with the radial sphincterotomy involving 2–3 incisions was carried out. In both groups: (a) no differentiation was made between patients regarding sex and age; (b) patients were confirmed to have no contraindications for sedation; (c) criteria for comorbidity were not implemented to form the groups.

### ERCP

A balloon extractor (Endoflex^®^; Olympus, Tokyo, Japan) was used to remove stones in 52 patients. Baskets were employed for stone removal (Hexanal^®^; Olympus) in two patients. For this purpose, 51 patients needed one session of ERCP, and three patients required two sessions.

ERCP was carried out under intravenous sedation. It was undertaken using a duodenoscope (TJF-150; Olympus) with a lateral field of view. After placing the patient in the left lateral position, a diagnostic upper gastrointestinal endoscopy was carried out in all cases before duodenoscope introduction. Postoperatively, patients were in the emergency room for 12 h, after which they were discharged home. Patients who suffered complications were in the emergency room for ≤24 h, after which they were discharged home. All were prescribed a semi-liquid diet.

### Description of the incision for a standard sphincterotomy

In group 1, in accordance with the accepted standard, a sphincterotomy incision was made in the direction of 11, 12 or 1 ‘O’ clock of a conventional clock depending on the anatomy of the papilla and stone size. The sphincterotomy was a standard “pull type” (Olympus Medical Systems, Tokyo, Japan).

**Figure 1:**
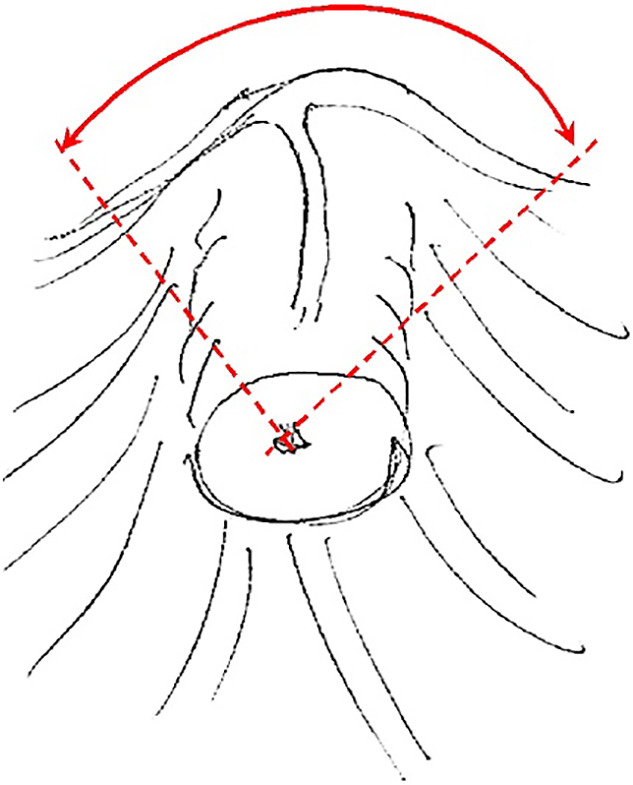
Safe incision area (schematic). Dashed lines indicate incision directions. A curved two-end arrow shows the opening angle of the incision.

The anatomy of the intraduodenal segment of the CBD and the large duodenal nipple permitted determination of the safety of the incision in the upper quarter of the nipple segment (Figure 1).

**Figure 2:**
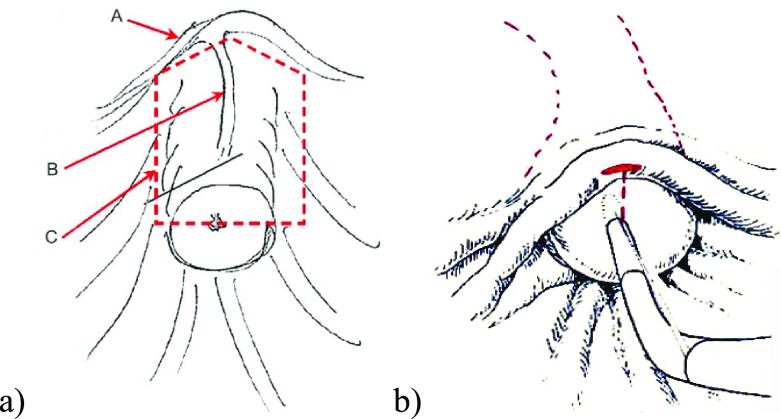
Schematic illustration of the (a) safe sphincterotomy area (A–plica transversa; B–plica longitudinalis; C–safe incision area) and (b) transverse dimension for the incision limit.

The maximum length of the incision was dependent upon the length of the longitudinal fold from the nipple to the first transverse fold to the nipple, and was up to 10–15 mm. Continuation of the incision above the groove for the transverse sinus is fraught with perforations, and the deviation from the interventricular groove can lead to perforations and bleeding. The safe boundary of the incision area of the sphincterotomy is shown in Figure 2.

**Figure 3:**
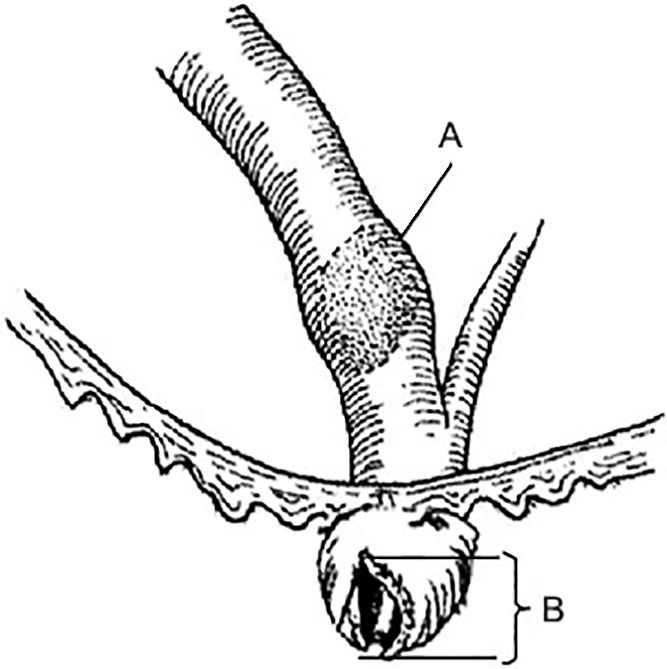
Incision for a standard sphincterotomy and large biliary stone: A) stone >20 mm, B) incision=15 mm

Thus, the natural safe anatomic limitations were around papillae, which made the standard-sphincterotomy incision insufficient in a case of large (>1.5 cm in diameter) or non-fissile (calcified) stone (Figure 3).

**Figure 4:**
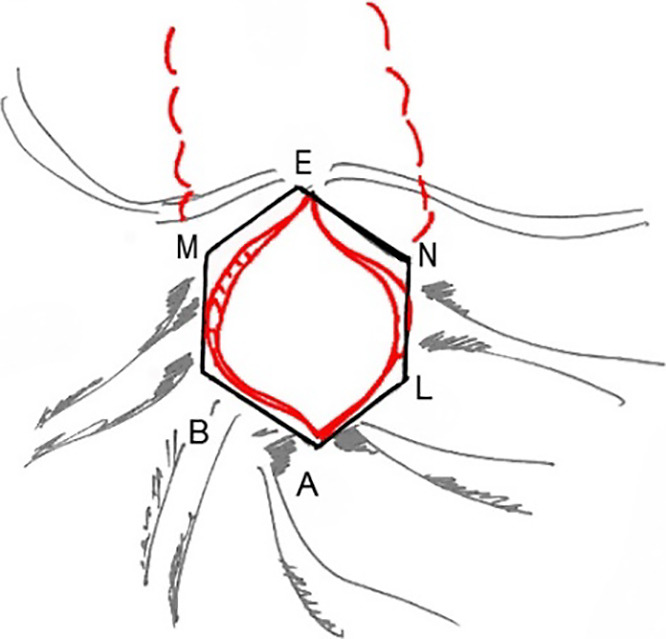
Maximum area in an incision for a standard sphincterotomy.

The maximum area provided by the incision for a standard sphincterotomy is shown in Figure 4.

### Description of the improved method for a radial sphincterotomy

**Figure 5:**
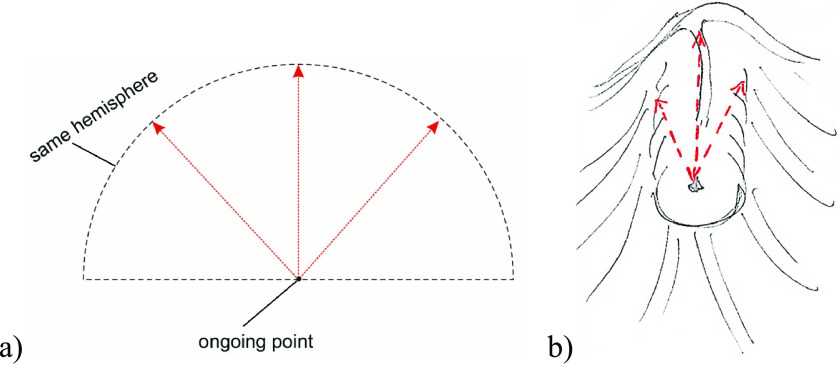
Incision direction in a radial sphincterotomy

The radial sphincterotomy we have developed makes it possible to carry out several incisions in a single session towards the 11, 12 and 13 ‘O’ clock of a conventional clock. Thus, the main incision can be made to the transverse fold, and other radial incisions should be made below the transverse fold, without going beyond the boundaries of the intended course of the intramural part of the CBD. The directions of the incisions of the radial sphincterotomy are shown in Figure 5.

**Figure 6:**
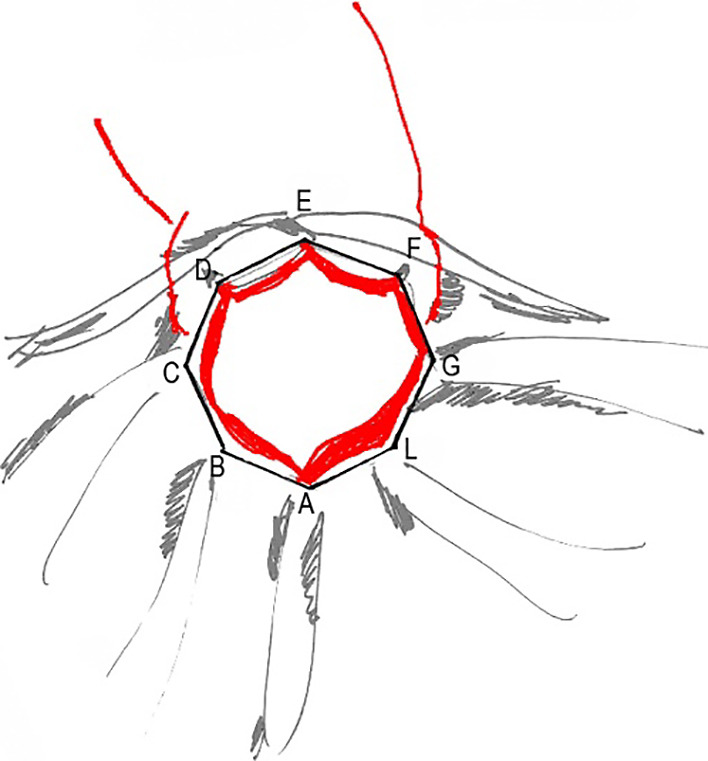
Maximum area in a radial sphincterotomy

The maximum area provided by the radial sphincterotomy is shown in Figure 6.

**Figure 7:**
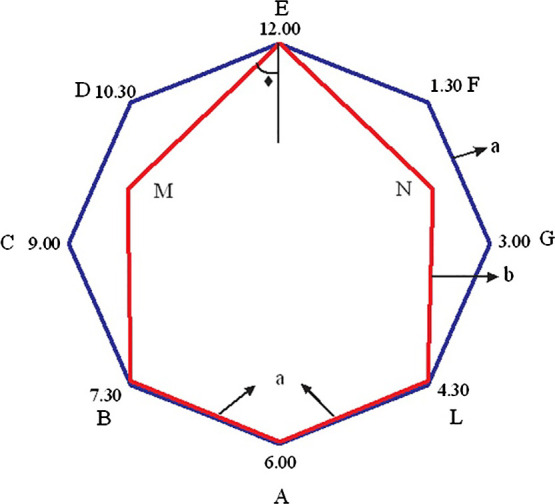
Comparison of the cross-sectional area between the standard sphincterotomy (ABMENL, shown in red) and radial sphincterotomy (ABCDEFGL, shown in blue).

Thus, the total cross-section of the incision with the additional cuts in the radial sphincterotomy became larger than the size of the main incision in the standard sphincterotomy. This scenario is evident from Figure 7, where the geometric shapes of the sections of the incisions of the standard sphincterotomy and radial sphincterotomy are compared schematically.

In the standard sphincterotomy, we assume that the cross-sectional area has an elongated (in the vertical direction) six-angle shape with the following definition:

(1) $$S_{6}=S_{\mathrm{ABMENL}}=S_{\mathrm{ABL}}+S_{\mathrm{BMNL}}+S_{\mathrm{MEN}}$$

In the radial sphincterotomy, the cross-section of the incision has the following area:

(2) $$S_{8}=S_{\mathrm{ABCDEFGL}}$$

To calculate the ratio of these areas,

(3) $$R=S_{8}/S_{6}$$

it is necessary to resort to mathematical calculations. Denoting the length of the AB segment as “a”, and the length of the BM segment as “b”, for the three terms in the right-hand side of equation (1) we have:

(4) $$S_{\mathrm{ABL}}=\frac{a^{2}}{2}\sin\left(2\beta \right)\text{,}\;S_{\mathrm{BMNL}}=2a\,b\cos\beta$$

(5) $$S_{\mathrm{MEN}}=ab\cos\beta \cos\gamma$$

where

(6) $$\beta =\frac{45}{2}\text{,}\;b=a\frac{\cos\beta }{\sin\gamma }$$

and the angle *β* is calculated from the following equation:

(7) $$\cot\beta =\frac{1}{\cos\beta }(\frac{1}{\sin\beta }-\sin\beta )-1$$

For the radial sphincterotomy-related area of the octagon in equation (2) we obtain:

(8) $$S_{8}=2a^{2}\frac{1}{\tan\beta }$$

Putting equations (4)–(8) into equation (3), we arrive at the final result for the efficiency factor R:

(9) R=1.48

or, in other words, the new size of the base of the papilla in the radial sphincterotomy becomes almost 1.5-times larger than in the standard section. Therefore, the radial sphincterotomy permits safely increasing the area of the base of the dissected papilla to remove large stones from the CBD.

## Results

In group 1, the stone size was <20 mm in 21 patients, and >20 mm in two patients. In group 2, stones >20 mm were detected in seven patients, and were 15–20 mm in other cases. Stone sizes were determined first by the results of ultrasound and MRI examinations for each patient. The data of measurements upon imaging were compared with the “true” size of the stones (measured by divisions within the lumen in the duodenum on the standard sphincterotomy) after removing them from the sphincterotomy incision. Stones >20 mm insusceptible to immediate removal were first crumbled with basket forceps, then removed with an extractor balloon. Stone size within the intestinal cavity was not measured, so only the size upon imaging was taken into account.

In both groups, the sphincterotomy incision was made taking into account the maximum size of the stone. If the stone size was >20 mm, it was first crushed, then removed in portions.In group 2, all patients underwent a main sphincterotomy incision along the main axis of the intramural section of the choledochus. Depending on the incision and shape of the papilla, and expression of the upper transverse fold (which determines the safe distance from the papilla opening to it), lateral radial incisions were made, thereby achieving an increase in the sphincterotomy incision as a whole.

The complications from ERCP were also documented. In group 1, the prevalence of pancreatitis was 4.3% (1/23) whereas in group 2 it was 3.2% (1/31). In group 1, the prevalence of bleeding was 8.7% (2/23), whereas no patients suffered bleeding in group 2.

The number of stones in group 1 was 3.39±1.3, and was 2.33±0.98 in group 2 (p=0.314). The stone size was 10.07±4.93 in group 1, and was 19.01±3.31 in group 2 (p=0.172).

All ERCP patients were observed during 3 years after surgery. No significant health complications related to ERCP and no new occurrences of stones in the CBD were recorded.

## Discussion

Removal of large stones (>20 mm) from the CBD, as well as the resulting complications, are important surgical issues.^10^

None of the patients in our study died, whereas studies have reported mortality of 2.2%–2.4% for CBD surgery.^4,5^ The common complications are intraoperative bleeding and postoperative pancreatitis. Our study also revealed the risk of perforation to be reduced to a minimum. We did not detect this complication, but other studies have reported the risk of perforation to be 0.56%–1.6%.^6,7^ If reactive pancreatitis occurred after contrast-medium administration, patients were prescribed a non-fat liquid diet and PPI (proton pump inhibitor) therapy. After 2–3 days, all symptoms of reactive pancreatitis disappeared.

There are four classifications of the major papilla: type 1 (regular papilla), type 2 (small papilla), type 3 (protruding or pendulous papilla) or type 4 (creased or ridged papilla).^11^ After standard sphincterotomy, approximately all CBDs are clear if stones are ≤10 mm. In many cases, stone passage occurs following endoscopic sphincterotomy immediately or after resolution of edema. However, clearing the CBD of stones is desirable to avoid acute cholangitis, which usually develops in patients with a retained stone after endoscopic sphincterotomy. A standard sphincterotomy based on cutting of the papilla is dependent upon the papilla type; it involves complete division of muscles in regular and protruding types, and a partial division of muscles in small and creased types, of papilla. In extensive sphincterotomy, the removal of large stones elicits damage to neighboring tissues. The actions described above increase the risk of duodenal perforations, including development of a periampullary diverticulum. To avoid such complications, we employed a radial sphincterotomy, which involves an uncompleted main incision cut with an additional two side-cuts in a safe area of the papilla.

Importantly, 29% (n=9) of patients who underwent a radial sphincterotomy had a periampullary diverticulum. The problem of ERCP and development of a periampullary diverticulum has been documented in recent years.^12,13^ The inadequate structure of the diverticulum wall compared with that of the normal intestinal wall can lead to complications (particularly perforations). Nevertheless, even in such cases, the radial sphincterotomy developed by our research team was successful, and intraoperative and postoperative complications were not observed. These findings demonstrated that this method was efficacious and safe for a periampullary diverticulum, but it should not be used if the diverticulum is 2-cm deep and, moreover, if the duodenal papilla is above the diverticulum. In these specific cases, optimal surgical access to the stone was lacking, which increased the perforation risk when carrying out the radial sphincterotomy. Also, the radial sphincterotomy should not be considered as an option for patients suffering from the secondary disease of an extrahepatic biliary tract. This condition results from a standard sphincterotomy conducted previously, which changes the anatomy of the surgical area.

Heo *et al.* described methods for removing large stones (>15 mm), as well as with an increase in the area of the dissected papilla, to facilitate stone passage.^14^ The essence of their method was to combine an endoscopic sphincterotomy and balloon dilatation of the papilla using mechanical lithotripsy. However, there are several problems when using this method. For example, after carrying out a balloon dilatation, the endoscopist must obtain the stone with a basket within a limited time, after which the sphincter will restore its size. Also, Heo and colleagues noted complications such as cholecystitis, which was not observed using our method.

Similar work was also carried out by Jin and coauthors (they observed complications such as hemorrhage), as well as by Yang and Hu (they documented bleeding and infection of the biliary tract).^15,16^ Jin *et al.* as well as Yang and Hu conducted a meta-analysis with a detailed and comprehensive endoscopic approach to stones in the CBD. Jin *et al.* did not detect a significant difference between endoscopic dilatation of the papilla using a large balloon *versus* endoscopic sphincterotomy for stones in the CBD.^15^ Those authors described the use and efficacy of endoscopic mechanical lithotripsy for large stones (15–20 mm), and the stones were removed after crushing. In our study, the radial sphincterotomy was carried out in a single session, which enabled avoidance of mechanical lithotripsy for large stones. There was a significant difference in the overall prevalence of adverse events (e.g., procedure-related pancreatitis or hemorrhage) in the studies by Jin *et al.* and Yang and Hu. Xu *et al.* noted that mechanical lithotripsy with an insufficient lumen size of the papilla created a risk of stone reformation after the sphincterotomy.^17^ Our radial sphincterotomy prevents this problem because the papilla diameter remains wide and, if stones are reformed, they fall freely into the intestinal lumen.

## Conclusions

We described a novel method, radial sphincterotomy, for ERCP to remove stones safely in the CBD without complications. The proposed method was justified from both anatomic and mathematical viewpoints. The increase in the area of the dissected papilla ensured safe removal of large stones without bleeding. Radial sphincterotomy deserves more extensive research and application. We recommend that only experienced endoscopists should carry out this novel procedure.
